# Supplementary material for: Neuronal functional connectivity is impaired in a layer dependent manner near chronically implanted intracortical microelectrodes in C57BL6 wildtype mice
Source: J Neural Eng. Author manuscript; Available in PMC 2025 Jun 7. (PMC11948186; doi:10.1088/1741-2552/ad5049)
Supplement: Supdoc [file NIHMS2036180-supplement-Supdoc.docx]

**Neuronal functional connectivity is impaired in a layer dependent manner near chronically implanted intracortical microelectrodes in C57BL6 Wildtype mice**

Keying Chen ^a, b^, Adam Forrest ^a, b^, Guillermo Gonzalez Burgos^c^, Takashi D.Y. Kozai ^a, b, d, e, f,*^

a, Department of Bioengineering, University of Pittsburgh, Pittsburgh, PA, USA

b, Center for Neural Basis of Cognition, Pittsburgh, PA, USA

c, Department of Psychiatry, University of Pittsburgh, Pittsburgh, PA, USA

d, Center for Neuroscience, University of Pittsburgh, Pittsburgh, PA, USA

e, McGowan Institute of Regenerative Medicine, University of Pittsburgh, Pittsburgh, PA, USA

f, NeuroTech Center, University of Pittsburgh Brain Institute, Pittsburgh, PA, USA

*, Corresponding Author: [TK.Kozai@pitt.edu](mailto:TK.Kozai@pitt.edu)

**Abstract**

*Objective:* This study aims to reveal longitudinal changes in functional network connectivity within and across different brain structures near the chronically implanted microelectrode. While it is well established that the foreign-body response (FBR) contributes to the gradual decline of the signals recorded from brain implants over time, how does the FBR impact affect the functional stability of neural circuits near implanted Brain-Computer Interfaces (BCIs) remains unknown. This research aims to illuminate how the chronic FBR can alter local neural circuit function and the implications for BCI decoders. *Approach:* This study utilized the single-shank, 16-channel,100 µm site-spacing Michigan-style microelectrodes (3mm length, 703 µm2 site area) that span all cortical layers and the hippocampal CA1 region. Sex balanced C57BL6 wildtype mice (11-13 weeks old) received perpendicularly implanted microelectrode in left primary visual cortex. Electrophysiological recordings were performed during both spontaneous activity and visual sensory stimulation. Alterations in neuronal activity near the microelectrode were tested assessing cross-frequency synchronization of LFP and spike entrainment to LFP oscillatory activity throughout 16 weeks after microelectrode implantation. *Main Results*: The study found that cortical layer 4, the input-receiving layer, maintained activity over the implantation time. However, layers 2/3 rapidly experienced severe impairment, leading to a loss of proper intralaminar connectivity in the downstream output layers 5/6. Furthermore, the impairment of interlaminar connectivity near the microelectrode was unidirectional, showing decreased connectivity from Layers 2/3 to Layers 5/6 but not the reverse direction. In the hippocampus, CA1 neurons gradually became unable to properly entrain to the surrounding LFP oscillations. *Significance*: This study provides a detailed characterization of network connectivity dysfunction over long-term microelectrode implantation periods. This new knowledge could contribute to the development of targeted therapeutic strategies aimed at improving the health of the tissue surrounding brain implants and potentially inform engineering of adaptive decoders as the FBR progresses. Our study's understanding of the dynamic changes in the functional network over time opens the door to developing interventions for improving the long-term stability and performance of intracortical microelectrodes.

**Keywords:** brain-computer interface, brain circuit, LFP synchronization, visual cortex, neuroinflammation.


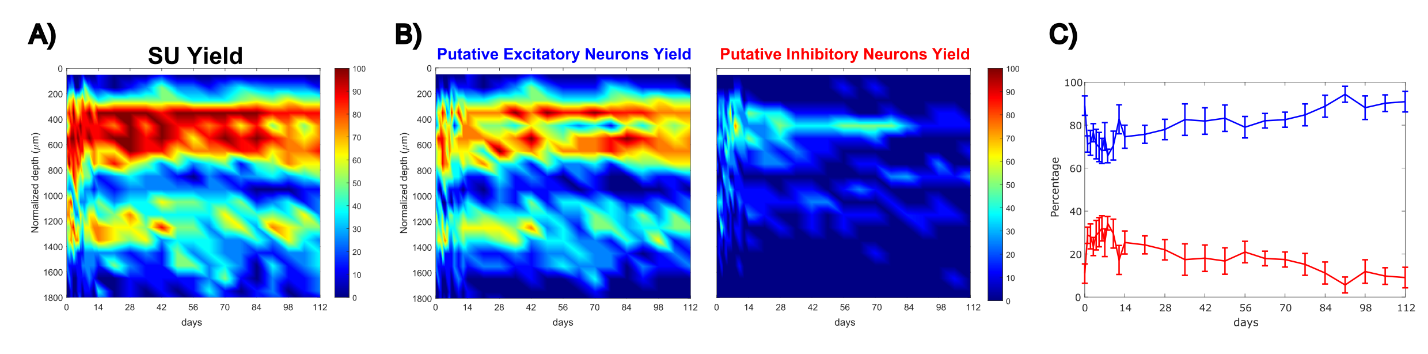


**Supplementary Figure 1: Reduced Single Unit (SU) detection occurred at chronic intracortical microelectrode implantation period.**  SU Yield was calculated as the percentage of channels that contain at least one SU. SU Yield (A) and putative excitatory, inhibitory neurons (B) was plotted as a function of depth and days post-implantation. C) Percentage of putative excitatory (blue) and inhibitory (red) neurons out of the total number of sorted SUs was plotted over time.


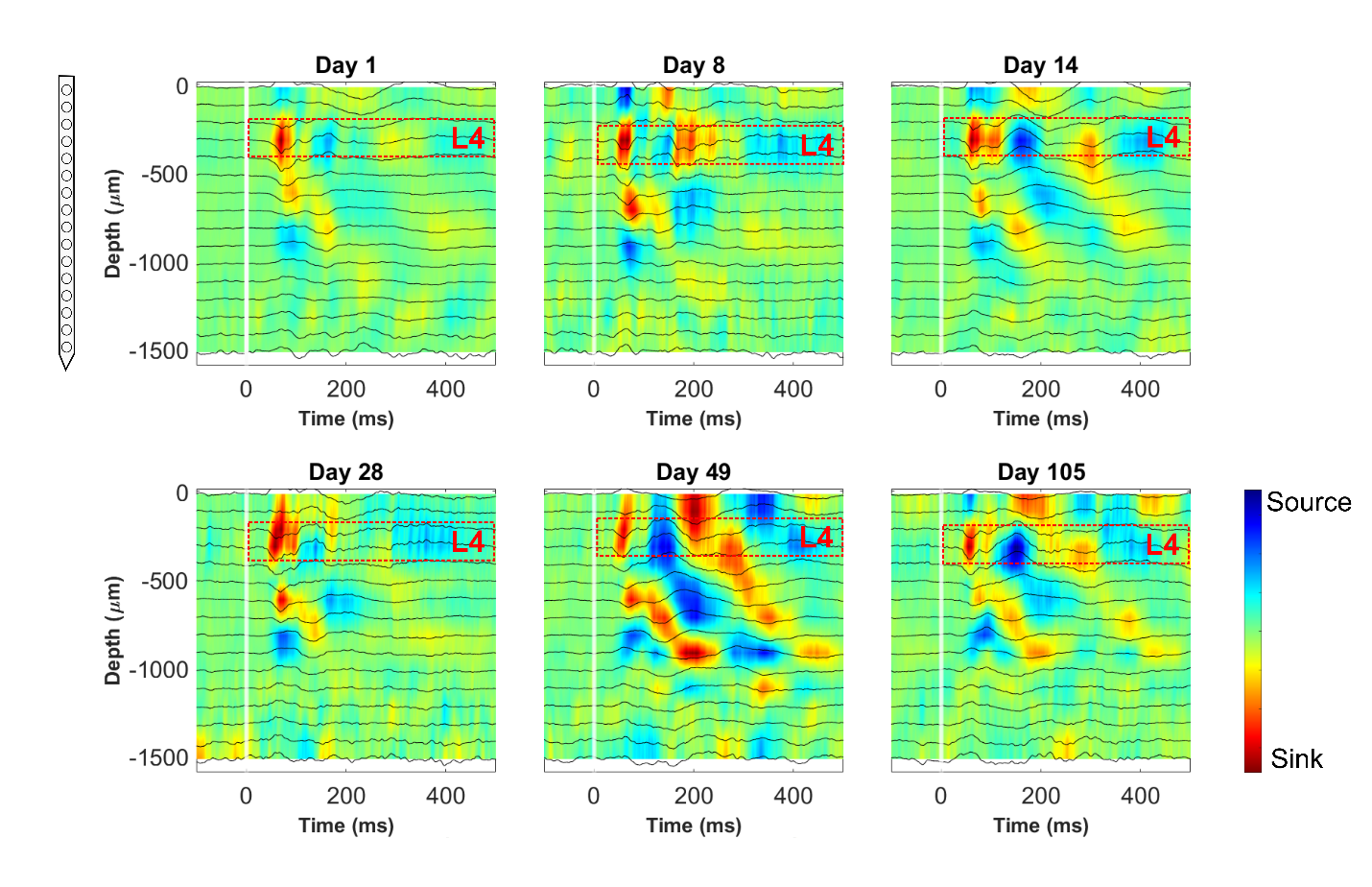


**Supplementary Figure 2: Time-Series of Current Source Density (CSD) Maps and Local Field Potential (LFP) Traces Following Visual Stimulation in a Representative Mouse**. The intracortical microelectrode was implanted in the primary visual cortex (V1). Layer 4 (L4) was identified based on the first source of LFP oscillation following the onset of visual stimuli.


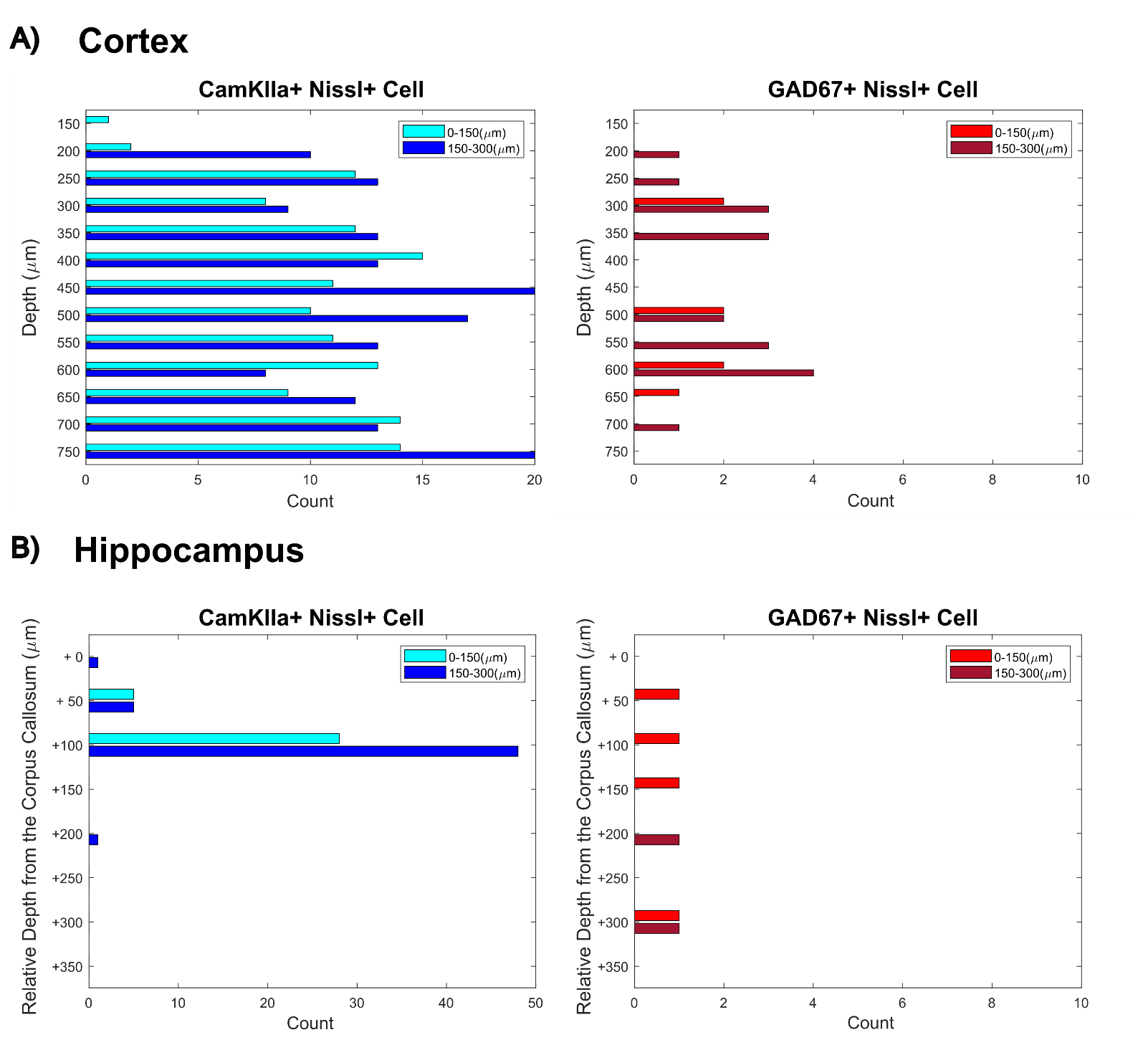


**Supplementary Figure 3: Depth profile of neuronal subtype populations near the microelectrode 16 weeks post-implantation.** Counts of Camkiia+ Nissl+ cells (left) and GAD67+ Nissl+ cells (right) within 150 μm and 150-300 μm away from the probe across different depth in cortex **(A)** and the hippocampal CA1 region **(B)**.

|  | Acute: Early Chronic | Early Chronic: Chronic | Acute: Chronic |
| --- | --- | --- | --- |
| L4 Putative Excitatory Neurons Entrainment to Intralaminar 2-7.5 Hz LFP | 0.142 | 0.386 | 0.361 |
| L4 Putative Inhibitory Neurons Entrainment to Intralaminar 2-7.5 Hz LFP | 0.279 | X | X |
| L4 Putative Inhibitory Neurons Entrainment to Intralaminar 80-90 Hz LFP | 0.725 | X | X |
| L2/3 Putative Excitatory Neurons Entrainment to Intralaminar 2-7.5 Hz LFP | 9.16 * 10^(-4) | 0.903 | 0.001 |
| L2/3 Putative Excitatory Neurons Entrainment to Intralaminar 60-90 Hz LFP | 3.56 * 10^(-5) | 0.203 | 0.015 |
| L5/6 Putative Excitatory Neurons Entrainment to Intralaminar 2-7.5 Hz LFP | 2.27 * 10^(-7) | 0.635 | 1.23 * 10^(-8) |
| L5/6 Putative Excitatory Neurons Entrainment to Intralaminar 60-90 Hz LFP | 0.048 | 0.263 | 3.49 * 10^(-4) |
| L5/6 Putative Inhibitory Neurons Entrainment to Intralaminar 2-7.5 Hz LFP | 5.00 * 10^(-6) | 0.391 | 1.19 * 10^(-6) |
| L5/6 Putative Inhibitory Neurons Entrainment to Intralaminar 60-90 Hz LFP | 0.012 | 0.812 | 0.085 |
| L2/3 Putative Excitatory Neurons Entrainment to L4 2-7.5 Hz LFP | 0.480 | 0.055 | 0.007 |
| L4 Putative Excitatory Neurons Entrainment to L2/3 2-20 Hz LFP | 1.96 * 10^(-6) | 0.599 | 1.36 * 10^(-7) |
| L2/3 Putative Excitatory Neurons Entrainment to L5 2-7.5 Hz LFP | 0.214 | 0.313 | 0.015 |
| L5 Putative Excitatory Neurons Entrainment to L2/3 2-20 Hz LFP | 3.42 * 10^(-6) | 0.232 | 2.78 * 10^(-5) |
| CA1 Putative Excitatory Neurons Entrainment to Intralaminar 2-7.5 Hz LFP | 0.104 | 0.722 | 0.039 |

**Supplementary Table 1**. Statistical analysis of Watson-Williams test for the resultant mean entrainment angles; significance *p*<0.05.

|  | Acute: Early Chronic | Early Chronic: Chronic | Acute: Chronic |
| --- | --- | --- | --- |
| L4 Putative Excitatory Neurons Entrainment to Intralaminar 2-7.5 Hz LFP | *p* > 0.1 | *p* > 0.1 | *p* > 0.1 |
| L4 Putative Inhibitory Neurons Entrainment to Intralaminar 2-7.5 Hz LFP | *p* < 0.02 | X | X |
| L4 Putative Inhibitory Neurons Entrainment to Intralaminar 80-90 Hz LFP | *p* > 0.1 | X | X |
| L2/3 Putative Excitatory Neurons Entrainment to Intralaminar 2-7.5 Hz LFP | *p* > 0.1 | *p* > 0.1 | *p* < 0.02 |
| L2/3 Putative Excitatory Neurons Entrainment to Intralaminar 60-90 Hz LFP | *p* < 0.02 | 0.05 < *p* < 0.1 | *p* > 0.1 |
| L5/6 Putative Excitatory Neurons Entrainment to Intralaminar 2-7.5 Hz LFP | *p* < 0.001 | *p* > 0.1 | *p* < 0.001 |
| L5/6 Putative Excitatory Neurons Entrainment to Intralaminar 60-90 Hz LFP | *p* > 0.1 | *p* > 0.1 | 0.05 < *p* < 0.1 |
| L5/6 Putative Inhibitory Neurons Entrainment to Intralaminar 2-7.5 Hz LFP | *p* < 0.001 | *p* > 0.1 | *p* < 0.005 |
| L5/6 Putative Inhibitory Neurons Entrainment to Intralaminar 60-90 Hz LFP | *p* > 0.1 | *p* < 0.05 | *p* < 0.005 |
| L2/3 Putative Excitatory Neurons Entrainment to L4 2-7.5 Hz LFP | *p* > 0.1 | *p* > 0.1 | 0.05 < *p* < 0.1 |
| L4 Putative Excitatory Neurons Entrainment to L2/3 2-20 Hz LFP | *p* < 0.001 | *p* > 0.1 | *p* < 0.001 |
| L2/3 Putative Excitatory Neurons Entrainment to L5 2-7.5 Hz LFP | *p* > 0.1 | *p* > 0.1 | *p* > 0.1 |
| L5 Putative Excitatory Neurons Entrainment to L2/3 2-20 Hz LFP | *p* < 0.02 | *p* > 0.1 | *p* < 0.001 |
| CA1 Putative Excitatory Neurons Entrainment to Intralaminar 2-7.5 Hz LFP | *p* < 0.02 | *p* > 0.1 | *p* > 0.1 |

**Supplementary Table** **2**. Statistical analysis of Kuiper test distribution of entrainment angles; significance *p*<0.05.
