# Supplementary figures and images for: Neuronal functional connectivity is impaired in a layer dependent manner near chronically implanted intracortical microelectrodes in C57BL6 wildtype mice
Source: J Neural Eng. Author manuscript; Available in PMC 2025 Jun 7. (PMC11948186; doi:10.1088/1741-2552/ad5049)

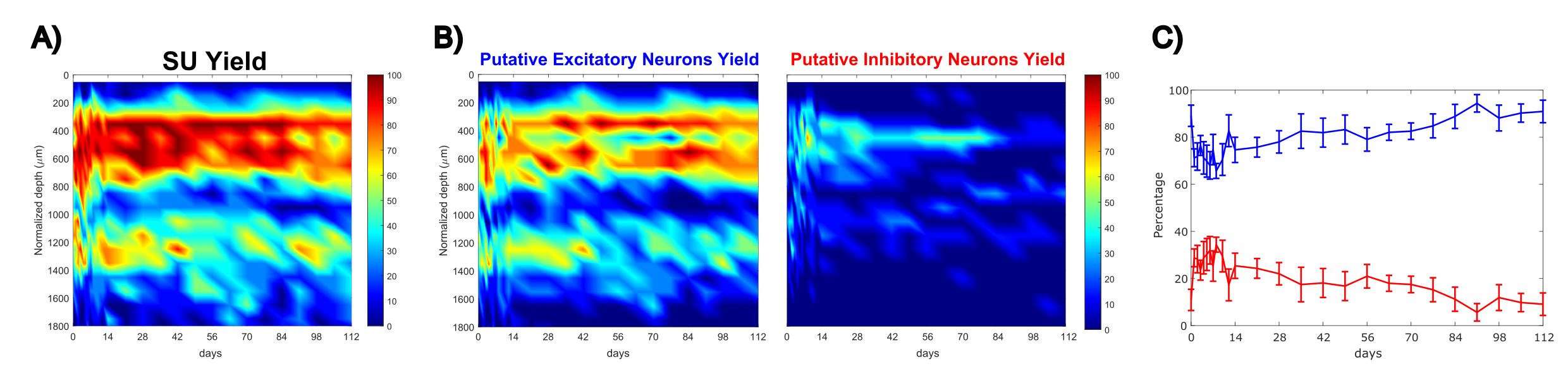

Supplement: SFIG1 [file NIHMS2036180-supplement-SFIG1.png]

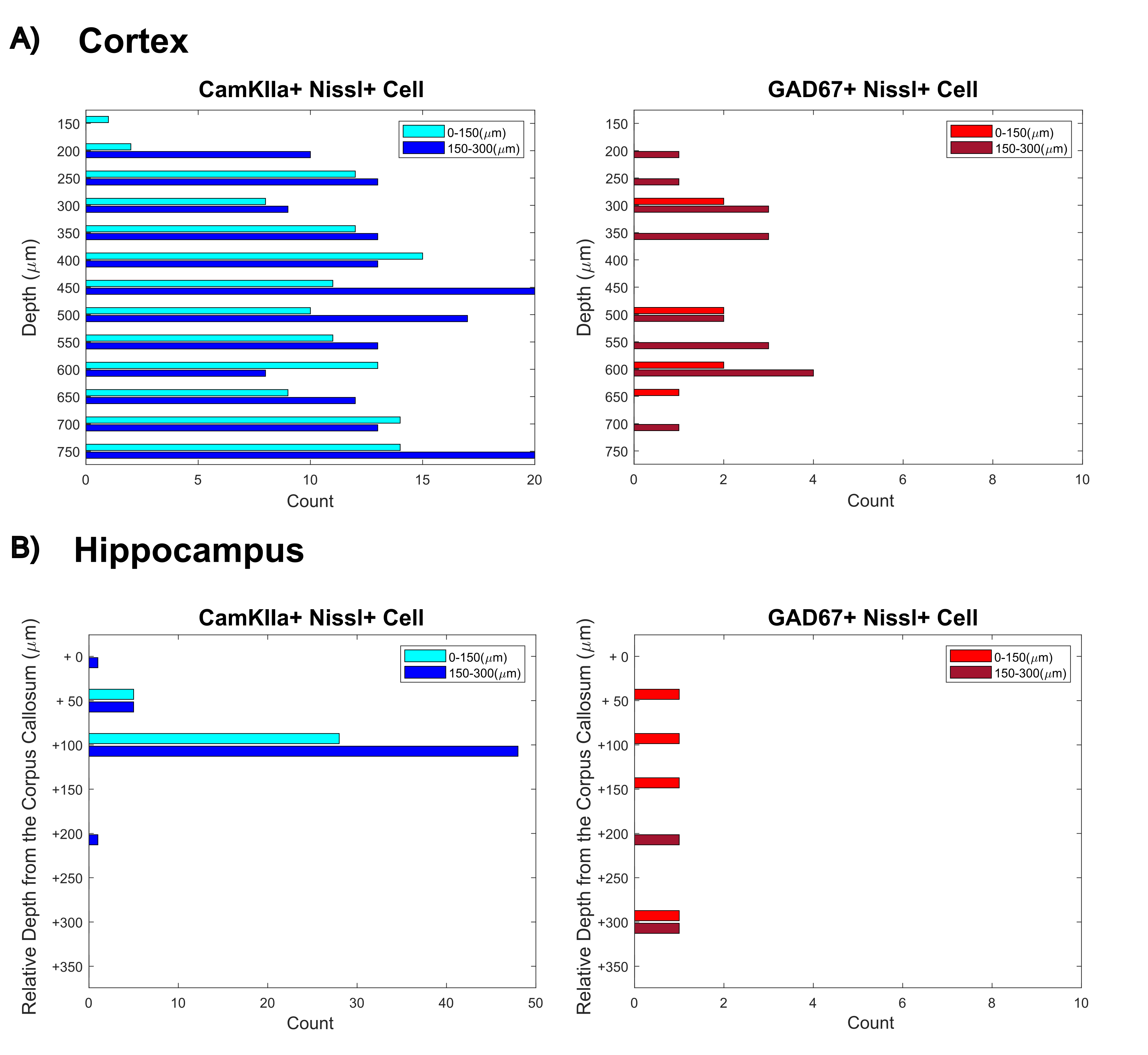

Supplement: Sfig3 [file NIHMS2036180-supplement-Sfig3.png]

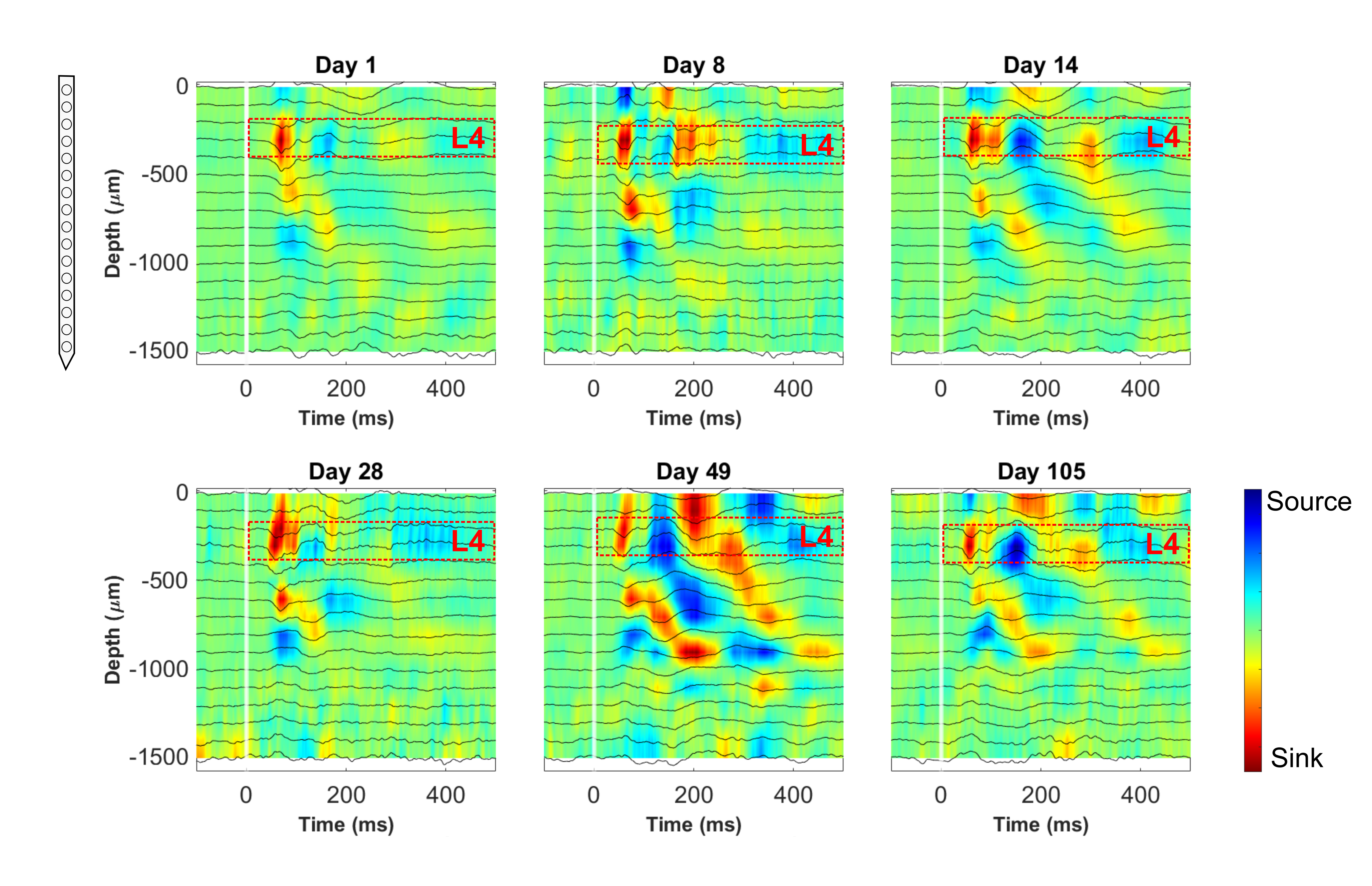

Supplement: SFig2 [file NIHMS2036180-supplement-SFig2.png]
